# A Pilot Study to Analyze the Relationship Between Left Ventricular Systole and Early Diastole: Clues Derived From Longitudinal Mitral Annular Motion

**DOI:** 10.7759/cureus.91029

**Published:** 2025-08-26

**Authors:** Khalid Sawalha, Aakash Rana, Angel Lopez Candales

**Affiliations:** 1 Cardiovascular Medicine, University of Arkansas for Medical Sciences, Little Rock, USA; 2 Cardiometabolic Medicine, University of Missouri Kansas City School of Medicine, Kansas City, USA; 3 Internal Medicine, Central Arkansas Veterans Healthcare System, Little Rock, USA

**Keywords:** left ventricular early diastolic function, left ventricular systolic function, mitral annulus, m-mode, tissue doppler imaging

## Abstract

Background: Cardiac rotation of the left ventricle (LV) along its long axis, due to the twisting motion of the double-loop helix orientation, has been shown to explain LV systolic and early diastolic function. However, the assessment of this phenomenon using cardiac magnetic resonance imaging and speckle-tracking echocardiography is not always available and has some limitations. Therefore, we aimed to simplify this assessment using M-mode and tissue Doppler imaging (TDI) interrogation of the mitral annulus (MA).

Methods: A total of 75 patients were studied and divided equally into three groups. Group 1 consisted of young patients with normal LV systolic function, Group 2 included older patients also with normal LV systolic function, and Group 3 comprised patients with reduced LV systolic function.

Results: As anticipated, MA excursion and TDI systolic velocities showed a strong correlation with LV systolic function. Notably, reduced LV systolic function was also associated with lower calculated MA early diastolic values. Moreover, early diastolic velocities were the only MA M-mode or TDI variable capable of distinguishing patients from all three studied groups. Finally, both MA systolic excursion and measured TDI velocities exhibited a significant correlation with both indices of LV systolic and early diastolic function.

Conclusion: Our data suggest that M-mode and TDI interrogation of the MA are not only useful in assessing LV systolic function, but also early LV diastolic function, as these two events are closely linked to longitudinal motion.

## Introduction

Cardiac rotation, a concept initially described by Leonardo da Vinci, continues to intrigue clinicians and researchers [1]. This cardiac torsion, measured as left ventricular (LV) twist or torsion, represents the mean longitudinal gradient of the net difference in clockwise and counterclockwise rotation of the LV apex and base, as viewed from the LV apex [2-5]. Twist during ejection predominantly deforms the subendocardial fiber matrix, resulting in the storage of potential energy, while the subsequent recoil of twist deformation is associated with the release of restoring forces, contributing to LV diastolic relaxation and early diastolic filling [2-5].

Despite the mechanical principles behind cardiac rotation, LV ejection fraction (LVEF) is still considered the most recognized variable when reporting systolic function on routine echocardiographic examinations [6]. Although LVEF results from the combined action of longitudinal and circumferential contraction, radial thickening, and basal to apical rotation, it has many limitations [7-9].

The heart's muscular structure, the myocardium, can be conceptualized as a single, coiled band that wraps around the heart like a helix. It forms two intertwined loops, resembling a double helix, with the apex of the heart acting as a vortex [10]. While the systolic LV rotation of the double helical structure of myocardial fibers could be conceptualized as the algebraic subtraction of the overall movement of the LV base toward the apex, resulting in longitudinal shortening of the LV that generates 60% of LVEF with only 15% fiber shortening [10], the resultant LV untwisting essentially constitutes diastolic filling [11,12]. Therefore, the longitudinal (basal to apical) excursion of the mitral annulus (MA) should also reflect LV systolic and diastolic functions [13-15].

Although two-dimensional speckle-tracking echocardiography has proven useful in assessing LV rotational motion [2,16,17], clinical studies have reported wide variability regarding resting LV systolic torsion values [18]. Nevertheless, echocardiographic speckle tracking remains promising due to its excellent temporal resolution [2,16-18]. However, cardiac magnetic resonance imaging (CMR) remains the indisputable gold standard imaging tool for assessing LV torsion [19,20]. 

Unfortunately, the limited availability of CMR clinical slots for specialized heart examinations and technical limitations, such as dependability on frame rate and image resolution, as well as the lack of routine use outside research applications, have markedly limited the widespread use of speckle-tracking echocardiography [21-25]. Furthermore, a significant number of patients are unable to undergo CMR examinations due to factors such as large body size, severe renal impairment, severe claustrophobia, or the presence of pacemakers and implantable cardioverter-defibrillators [21]. 

In contrast, M-mode echocardiography, known for its superb temporal imaging resolution, is easy to perform and does not require any offline algorithms or special imaging acquisition sequences [26]. Therefore, M-mode echocardiography appears as a valuable imaging tool to study the relationship between LV systolic and early diastolic function. Building upon our previous work in describing the anatomical and functional differences between tricuspid and mitral annular (MA) motion in terms of the systolic assessment of each respective ventricle, we have now decided to investigate whether MA motion might provide useful information regarding the relationship between LV systolic and early diastolic function [27,28]. This study aimed to ascertain the relationship between MA systolic dynamics and LV diastolic function parameters utilizing M-mode and tissue Doppler imaging (TDI). We posited that these two phenomena are closely interconnected, and thus, MA longitudinal motion may serve as a valuable indicator in evaluating early LV diastolic function.

## Materials and methods

For this study, we reviewed our echocardiographic database echocardiograms conducted between August 2019 and February 2020 on patients referred to our echocardiography laboratory at the University of Arkansas for Medical Sciences (Little Rock, AR, USA). The echocardiographic database was queried for complete transthoracic echocardiograms that included the acquisition of mitral valve inflow Doppler signals, TDI, as well as M-mode interrogation of the lateral MA. Additionally, all patients had to be in normal sinus rhythm at the time of the examination without any ectopic beats. Patients with sinus tachycardia were excluded. Echocardiograms were selected from patients with comparable body surface area (BSA) for the purpose of this study. The study population was divided into three groups: Group 1 comprised young patients with normal LV systolic function, Group 2 included older patients also with normal LV systolic function, and Group 3 consisted of patients with reduced LV systolic function. The study cohort comprised individuals with preserved LV systolic function across varying age groups to account for age-related variability, whereas Group 3 specifically compared participants with reduced versus preserved systolic function.

Study approval was obtained from the University of Arkansas for Medical Sciences Institutional Review Board.

Echocardiographic studies

Two-dimensional echocardiographic studies were performed using commercially available systems (Vivid 7 and 9; GE Medical Systems, Milwaukee, WI). Images were obtained in the parasternal and apical views with the patient in the left lateral decubitus position and in the subcostal view with the patient in the supine position using a 3.5 MHz transducer. Standard two-dimensional, color, pulsed, and continuous-wave Doppler data were digitally acquired during gently held end-expiration and saved in regular cine loop format for subsequent offline analysis.

The following parameters were measured to accomplish the goals of this study:

A) LV end-systolic and end-diastolic volumes were traced from the apical four-chamber view in accordance with published data. Ejection fraction calculations were done using the Simpson’s rule algorithm [6].

B) Mitral inflow velocities were obtained using pulsed-wave Doppler examination at a sweep speed of 100 mm/s from the apical four-chamber view. The sample volume was placed at the tips of the mitral leaflets, and peak early diastolic (E-wave) velocity was measured as previously described [29].

 C) TDI of the lateral portion of the MA was performed to obtain systolic, early diastolic (E′), and late diastolic (A′) velocities [29,30]. This was accomplished by placing the sample volume at the junction where the mitral valve plane intersects the LV free wall using images obtained from the apical four-chamber view. LV diastolic pressures were estimated using the mitral valve inflow E/MA TDI E′ ratio [29].

 D) Total MA excursion was obtained by placing the M-mode cursor at the junction of the MA plane with the LV free wall, allowing maximal displacement of the MA from its highest position after atrial ascent to the maximal descent during ventricular systole, as measured from the apical four-chamber view [27,28]. MA ascent (MAa) was measured as the distance traversed by the MA from the end of diastasis until the end of atrial contraction [27,28]. The early diastolic MA component was obtained by subtracting MAa from MA peak systolic excursion (MAPSE) [27,28]. 

**Figure 1:**
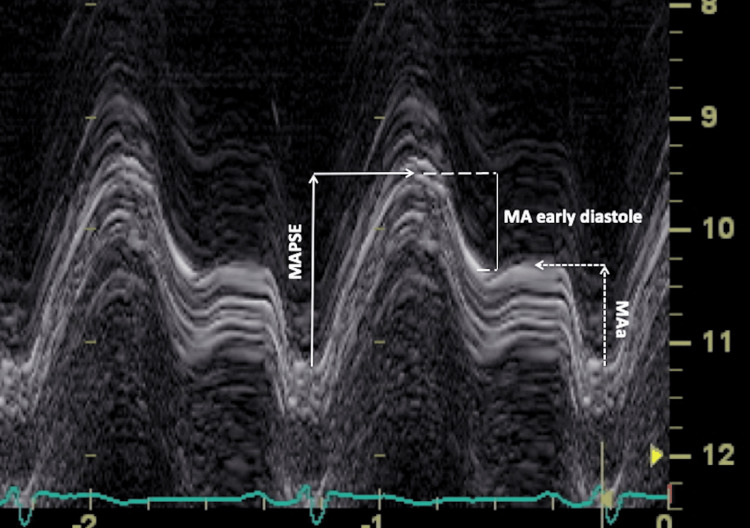
MA M-mode image showing MAPSE, MAa, and the calculated early diastolic MA component. MA, mitral annular; MAPSE, mitral annular plane systolic excursion; MAa, mitral annular ascent.

A representative MA M-mode image showing MAPSE, MAa, and the calculated early diastolic MA component is shown in Figure 1.

Statistical analysis

All echocardiographic parameters were calculated using the commercially available software Merge Cardio Workstation (Merge Healthcare, Chicago, IL).

Baseline characteristics were compared between groups using analysis of variance (ANOVA) for continuous variables. Echocardiographic measurements were compared using a two-tailed unpaired t-test assuming unequal variances. A stepwise multiple linear regression analysis was performed to determine the independent relationship between MAPSE and all significant echocardiographic variables found in the univariate analysis. A similar analysis was then performed for MA TDI early diastolic velocity. p-Values of <0.05 were considered statistically significant.

All statistical calculations were done using MedCalc Software bvba Version 14.12.0 (MedCalc Software Ltd, Ostend, Belgium).

## Results

A complete transthoracic echocardiogram with adequate endocardial border resolution to allow determination of end-diastolic and end-systolic LV volumes, as well as MA TDI and M-mode interrogation, was obtained in 75 consecutive patients from our echocardiographic database.

This population was divided as described in the Methods section: Group 1 comprised a total of 25 young patients with normal LV systolic function (17 men, mean age 36 ± 7 years, and BSA of 2.0 ± 0.4); Group 2 included 25 older patients with normal LV systolic function (13 men, mean age 59 ± 5 years, and BSA of 2.1 ± 0.2); and Group 3 consisted of 25 patients with reduced LV systolic function (12 men, mean age 56 ± 14 years, and BSA of 1.9 ± 0.3).

All echocardiographic parameters are listed in Table 1.

**Table 1:** Echocardiographic data for the studied population. Group 1 is statistically different from Groups 2 and 3 (p < 0.05). Group 3 is statistically different from Groups 1 and 2 (p < 0.05). Group 2 is statistically different from Groups 1 and 3 (p < 0.05). MAPSE, mitral annular plane systolic excursion; MV E/A ratio, mitral valve early-to-atrial diastolic flow velocity ratio; MV E/MA E′ ratio, mitral valve early diastolic velocity to mitral annular early diastolic tissue Doppler velocity ratio; MA S′ velocity, mitral annular systolic tissue Doppler velocity.

Variables	Group 1	Group 2	Group 3	ANOVA
LV mass index	99 ± 48	94 ± 31	130 ± 45	0.013
LV end-systolic volume	41 ± 15	32 ± 14	128 ± 59	<0.001
LV end-diastolic volume	130 ± 38	116 ± 36	181 ± 61	<0.001
LV ejection fraction	69 ± 6	73 ± 9	32 ± 12	<0.001
MAPSE	1.4 ± 0.2	1.4 ± 0.2	0.8 ± 0.3	<0.001
MV E/A ratio	1.4 ± 0.7	1.0 ± 0.4	2.1 ± 1.9	0.011
MV E/MA E’ ratio	9 ± 5	11 ± 8	18 ± 10	<0.001
MA S’ velocity	9.1 ± 2.7	8.4 ± 2.3	5.6 ± 2.0	<0.001

As expected, statistically significant differences were noted in all the recorded echocardiographic variables when comparing Group 3 with both Groups 1 and 2, except for the mitral valve inflow E/A ratio. The E/A ratio was found to be lower in Group 2 patients, but there was no difference in the MV E/A ratio between Group 1 and Group 3 patients.

**Figure 2:**
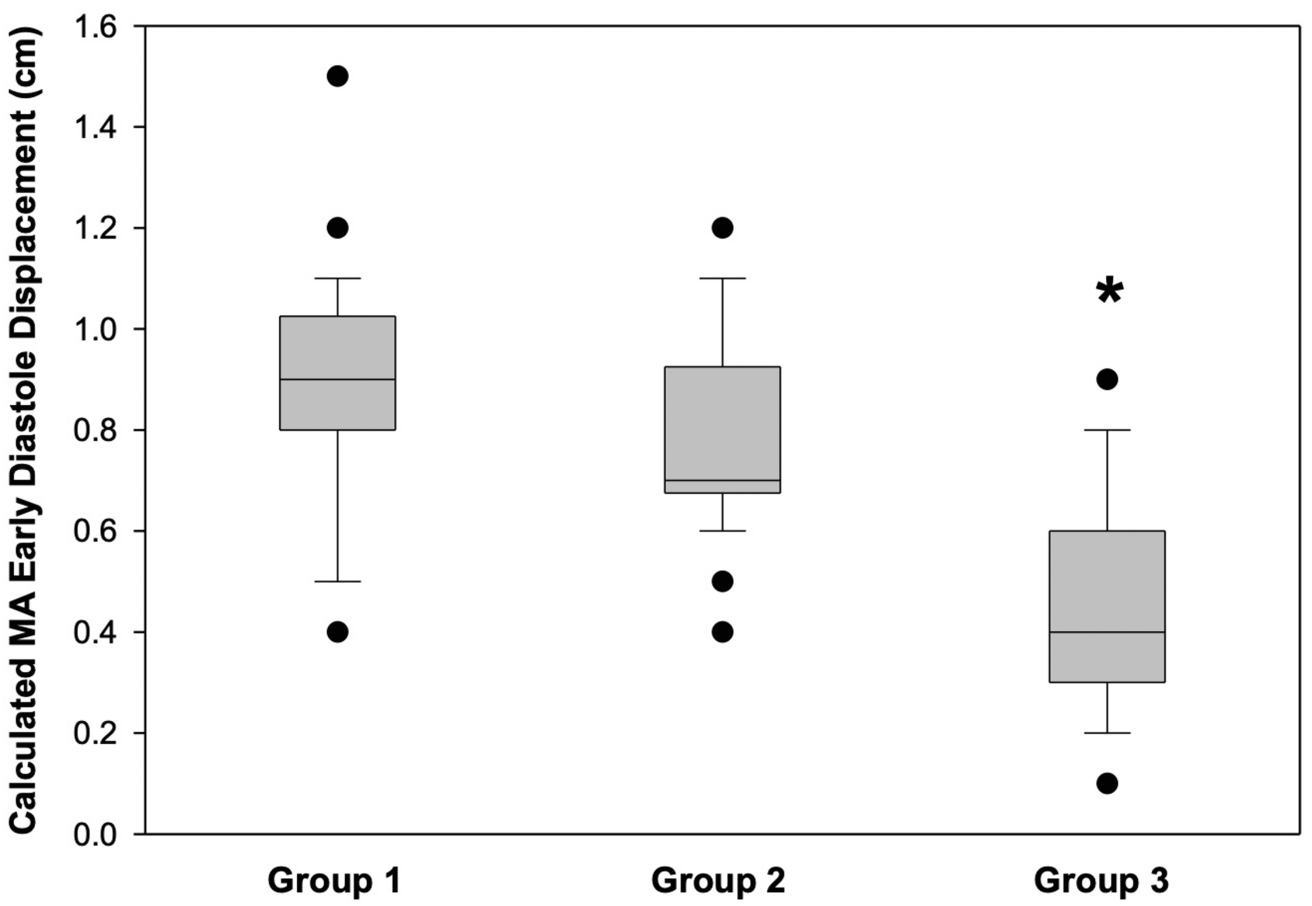
Box plot illustrating the calculated early diastolic displacement of MA in all three studied groups. *p < 0.05; statistically significantly different from Groups 1 and 2. MA, mitral annulus.

Regarding MAPSE and MAa measurements, we found no statistical difference between Group 1 (1.4 ± 0.2 cm and 0.5 ± 0.2 cm) and Group 2 (1.4 ± 0.2 cm and 0.6 ± 0.2 cm) patients. However, as expected, both measurements were significantly lower in Group 3 (0.8 ± 0.3 cm and 0.3 ± 0.2 cm; p < 0.001) patients. Consequently, as seen in Figure 2, significant differences were observed in terms of the calculated MA early diastole component.

**Figure 3:**
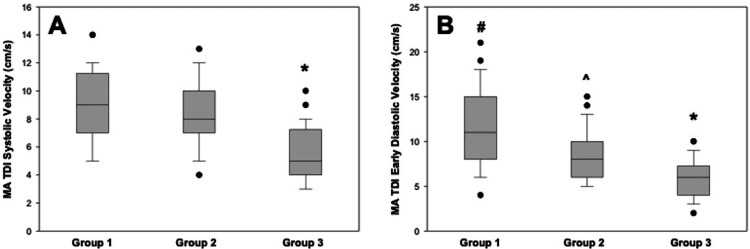
Box plot showing (A) MA TDI systolic velocities (*p < 0.05; statistically significantly different from groups 1 and 2) and (B) early diastolic velocities (#p < 0.05; statistically significantly different from groups 2 and 3; ^p < 0.05; statistically significantly different from groups 1 and 3; and *p < 0.05; statistically significantly different from groups 1 and 2). MA TDI, mitral annular tissue Doppler imaging.

We then compared MA TDI systolic and early diastolic velocities among the studied groups (Figure 3A) and found that, as expected, Group 3 patients had statistically lower MA TDI systolic velocity compared to both Group 1 and 2 patients. However, in terms of MA TDI early diastolic velocities, the highest velocities were observed in Group 1, with intermediate values for Group 2, and the lowest values recorded in Group 3 patients (Figure 3B).

A stepwise multiple regression analysis was performed to determine which of the measured echocardiographic variables correlated with MAPSE (Table 2). A similar analysis was conducted to determine which echocardiographic variables best correlated with MA TDI early diastolic velocity (Table 3).

**Table 2:** Stepwise multiple regression analysis showing which of the measured echocardiographic variables correlated with MAPSE. LVEF, left ventricular ejection fraction; MA TDI, mitral annular tissue Doppler imaging; MAPSE, mitral annular plane systolic excursion.

Independent variables	Coefficient	Standard error	p-Value
LVEF	0.007163	0.001018	<0.0001
MA TDI systolic velocity	0.02897	0.009194	0.0024
MA TDI early diastolic velocity	-0.01768	0.006829	0.0117
Calculated MA early diastole	0.7332	0.09127	<0.0001

**Table 3:** Stepwise multiple regression analysis showing which of the measured echocardiographic variables correlated with MA TDI early diastolic velocity. MAPSE, mitral annular plane systolic excursion; MA TDI, mitral annular systolic tissue Doppler velocity.

Independent variables	Coefficient	Standard error	p-Value
MAPSE	-3.4448	1.4975	0.0244
MA TDI systolic velocity	0.7706	0.1384	<0.0001
Calculated MA early diastole	9.1521	1.7523	<0.0001

**Figure 4:**
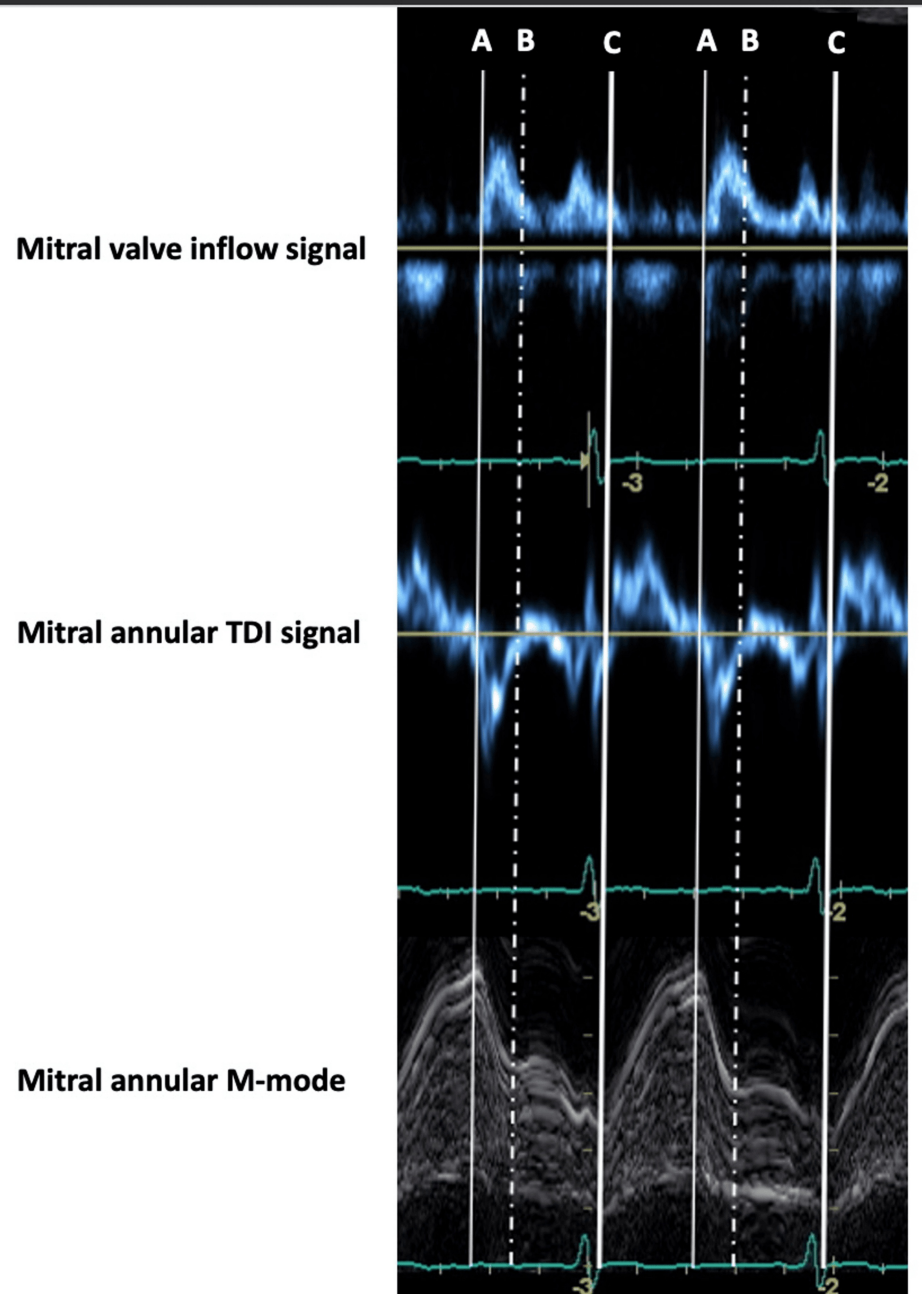
Representative images of mitral inflow, mitral annular TDI, and M-mode from the same patient, showing the correlation among cardiac events as they are individually depicted in each imaging modality. Two cardiac cycles are shown. (A) Thin white line indicates the onset of LV diastole, (B) dashed white line corresponds to the end of early LV diastole, and (C) thick white line identifies the onset of LV systole. TDI, tissue Doppler imaging; LV, left ventricle.

A representative set of mitral inflow, MA TDI, and M-mode images from the same patient, showing the correlation between these three cardiac events as individually inscribed for each imaging modality, is shown in Figure 4.

## Discussion

To the best of our knowledge, this is the first study directly aimed at using MA TDI and M-mode imaging to assess the potential utility of these imaging tools in routine echocardiography for evaluating the functional and mechanical link between LV systolic and early diastolic function.

Our study confirmed the strong relationship between LV systolic function and MAPSE, as well as MA TDI systolic velocities, as previously reported [6,27-29]. However, we have made several novel findings in this study. First, reduced LV systolic function is associated not only with lower MAa measurements but also with lower calculated MA early diastolic values compared to both young and old patients with normal LV systolic function. Secondly, early diastolic velocities were the only MA M-mode or TDI variable that could distinguish patients from all three studied groups. Finally, while LV systolic function showed the best correlation with the calculated MA early diastolic component, the MA TDI systolic velocity exhibited the best correlation with the calculated MA early diastolic component.

From a mechanistic standpoint, it is important to consider that in early diastole, LV relaxation reveals stored elastic strain, allowing the LV to recoil and act as a suction pump by rapidly drawing blood into the LV [31]. This close coupling of LV filling and rapid recoil generates an asymmetric toroidal vortex [32]. This toroidal vortex, which is defined by the length-to-diameter ratio equivalent to a cylindrical fluid column system, is assisted by the resultant atrioventricular pressure gradient and the MA serving as a piston for transmitral flow and vortex formation [32]. Our findings support the concept of the MA piston, as demonstrated by the usefulness of MAPSE and the MA early diastolic component in examining the close relationship between LV systolic and early diastolic function mechanics in a longitudinal assessment. Furthermore, the motion of the MA should reflect the overall mechanical function of the LV, as supported by a previous work by Morris et al., which found that LV global longitudinal systolic strain and diastolic strain rate were important predictors of left atrial longitudinal systolic and diastolic functions [33]. These results align with our findings since both LV and left atrial function are implicitly reflected in MA motion [27-29]. Additionally, our MA data is consistent with the work of van Dalen et al., which demonstrated the close relationship between the parabolic shape of the LV, twisting, and MA excursion throughout the cardiac cycle [34].

There are limitations to our study. First, the sample size was small, but this was a pilot study intended to demonstrate the proof of concept, which we achieved. Secondly, we did not perform concurrent LV speckle-tracking imaging, although we have previously correlated MAPSE with speckle-tracking data using automated functional imaging software [35]. Thirdly, we did not investigate the influence of arrhythmia or frequent ectopy on MA motion or the interpretation of LV systole and early diastole. Finally, although we did not measure left atrial volume or function in this study, as they could be altered in cases of LV systolic and diastolic dysfunction, an alternative approach of measuring the mitral valve E/MA E' ratio, which correlates well with mean pulmonary capillary wedge pressure and LV filling pressures, could have been sufficient [30].

## Conclusions

Our study demonstrates the potential utility of M-mode and TDI imaging techniques in routine echocardiography for evaluating the relationship between LV systolic and early diastolic function. The findings highlight the importance of MA motion in reflecting LV mechanical function and its close coupling with LV filling dynamics. This study suggests that MA assessment can provide valuable insights into LV systolic and diastolic function, especially when more advanced imaging techniques like CMR are not readily available or feasible. The results warrant further investigation and validation in larger studies to establish the clinical significance of MA assessment in routine echocardiographic examinations.
